# Salusins in Atherosclerosis: Dual Roles in Vascular Inflammation and Remodeling

**DOI:** 10.3390/biomedicines13081990

**Published:** 2025-08-15

**Authors:** Leszek Niepolski, Szymon Jęśko-Białek, Joanna Niepolska, Agata Pendzińska

**Affiliations:** 1 Faculty of Medicine, Prince Mieszko I Medical Academy in Poznan, Bułgarska 55, 60-321 Poznan, Poland; szyjes@st.amu.edu.pl; 2 Faculty of Medicine, Poznan University of Medical Sciences, Fredry 10, 61-701 Poznan, Poland; a.niepolska@gmail.com; 3 Department of Pathomorphology and Clinical Immunology, Poznan University of Medical Sciences, Przybyszewskiego 49, 60-355 Poznan, Poland; agatab@ump.edu.pl

**Keywords:** atherosclerosis, salusin-α, salusin-β, vascular inflammation

## Abstract

Atherosclerosis is a multifactorial, chronic inflammatory disorder characterized by the progressive accumulation of plaque within the arterial wall. Recent research has highlighted the pivotal role of bioactive peptides in modulating vascular homeostasis and inflammation. Among these, salusin-α and salusin-β have emerged as critical regulators of atherogenesis. These peptides are generated via differential proteolytic processing of preprosalusin: an amino acid precursor encoded by the torsin family 2 member A gene. Despite their common origin, salusin-α and salusin-β exhibit divergent biological activities. Salusin-β promotes vascular inflammation by enhancing oxidative stress, activating the nuclear factor kappa B signaling pathway, and upregulating proinflammatory cytokines as well as adhesion molecules, and it also facilitates foam cell formation by increasing the expression of acyl-CoA/cholesterol acyltransferase 1 and scavenger receptors, thereby contributing to plaque progression. In contrast, salusin-α appears to exert protective, anti-inflammatory, and anti-atherogenic effects by increasing the expression of the interleukin-1 receptor antagonist and inhibiting key proinflammatory mediators. Additionally, these peptides modulate the proliferation of vascular smooth muscle cells and fibroblasts, with salusin-β promoting cellular proliferation and fibrosis via calcium and 3′,5′-cyclic adenosine monophosphate-mediated pathways, while the role of salusin-α in these processes is less well defined. Altered plasma levels of salusins have been correlated with the presence and severity of atherosclerotic lesions, suggesting their potential as diagnostic biomarkers and therapeutic targets. This review provides a comprehensive overview of biosynthesis, tissue distribution, and dual roles of salusins in vascular inflammation and remodeling, emphasizing their significance in the pathogenesis and early detection of atherosclerotic cardiovascular disease.

## 1. Introduction

Atherosclerosis is a chronic inflammatory condition marked by the progressive accumulation of plaque in the arterial wall. Recent evidence underscores the pivotal role of bioactive peptides in regulating vascular homeostasis and inflammatory processes. Among these, salusin-α and salusin-β—both derived from a shared precursor—demonstrate opposing effects on atherogenesis: salusin-β promotes inflammation and accelerates the progression of atherosclerotic changes, whereas salusin-α confers vascular protection. Despite increasing interest in research, the distinct functional roles and differences between salusin-α and salusin-β in atherogenesis are still not fully elucidated. This review explores the biosynthesis, tissue distribution, and potential of sulusins as diagnostic biomarkers and therapeutic targets in atherosclerotic cardiovascular disease.

## 2. Biosynthesis and Tissue Expression of Salusins

Salusins are multifunctional bioactive peptides that were first described by Shichiri et al. [1] in 2003. Their polypeptide precursor, preprosalusin, is produced via alternative exon splicing of the gene encoding the protein known as torsin family 2 member A and consists of 242 amino acid residues. The transcriptional and epigenetic regulation of preprosalusin expression remains incompletely understood. Studies utilizing human neuroblastoma cells have demonstrated that expression of the preprosalusin gene and prosalusin protein is upregulated under serum deprivation conditions. Notably, inhibition or knockdown of the Jak-2 signaling pathway results in increased preprosalusin expression, implicating Jak-2 as a negative regulator of its transcription [2]. To date, there is no direct evidence supporting epigenetic regulation of salusin expression. Cleavage of the N-terminal signal peptide of preprosalusin yields prosalusin: a 216-amino acid protein. Subsequently, two active peptides are cleaved from the C-terminal region of prosalusin: salusin-α, which is composed of 28 amino acids, and salusin-β, which is composed of 20 amino acids [1]. These peptides differ in their hydrophobic amino acid content; salusin-β contains a higher proportion of hydrophobic residues, which confers unique physicochemical properties [1,3]. Notably, salusin-β adheres readily to polypropylene, polystyrene, and glass laboratory tubes, thereby significantly complicating experimental investigations [4,5]. These physicochemical differences may not only hinder experimental handling but could also influence their divergent biological effects. Both salusin isoforms are detected in body fluids and across various tissues. They are highly expressed in the cells of blood vessels, the nervous system, kidneys, stomach, lungs, liver, and bone marrow in particular [5,6,7,8]. Plasma concentrations of salusins vary and are influenced by multiple factors, whereas the urinary concentration of salusin-α is approximately tenfold lower than that of salusin-β [5,8]. Recent findings suggest that various pathological conditions can significantly affect plasma levels of these peptides. Consequently, numerous clinical studies have sought to elucidate the relationships between plasma salusin concentrations and specific disease states [9,10]. Their association with metabolic disorders and certain neoplastic diseases is of particular interest. For instance, Argun et al. demonstrated that patients with type 2 diabetes exhibit significantly reduced plasma levels of salusin-α and elevated levels of salusin-β compared to non-diabetic individuals [10]. Moreover, increased expression of salusin-α has been observed in ovarian cancer cells and certain cell lines derived from monoblastic leukemia [9,11]. Its broad tissue distribution and disease-specific variability in plasma levels suggest that salusins may serve as valuable biomarkers or molecular targets in metabolic and neoplastic conditions.

## 3. Influence of Salusins on Inflammatory Processes in Atherosclerotic Plaque

Atherosclerosis is currently regarded as a chronic inflammatory disease with an autoimmune component that develops within the arterial wall [12,13]. Endothelial dysfunction is a fundamental early event in the pathogenesis of atherosclerosis [14,15]. Both in vitro and in vivo studies indicate that salusin-β intensifies inflammatory processes in vascular endothelial cells [16]. By increasing oxidative stress, salusin-β stimulates the nuclear factor kappa B signaling pathway, which subsequently induces inflammatory processes in endothelial cells [17]. Furthermore, salusin-β enhances the expression of adhesion molecules, specifically vascular cell adhesion molecule-1 (VCAM-1), and the mRNA encoding intercellular adhesion molecule-1 (ICAM-1) [16,18]. It has also been demonstrated that salusin-β upregulates the expression of interleukin-1β (IL-1β), interleukin-6 (IL-6), interleukin-8 (IL-8), interleukin-18 (IL-18), and tumor necrosis factor-α (TNF-α) [3,16,18,19]. These cytokines contribute to the development of atherosclerosis and exacerbate inflammation within the vascular wall [13]. Recent studies have further shown that salusin-β elevates the level of monocyte chemoattractant protein-1 (MCP-1) [3,15,18], a chemokine with proinflammatory properties, that promotes the recruitment of macrophages and monocytes to sites of inflammation or atherosclerotic lesions [13,20]. Additionally, research by Esfahani et al. demonstrated that salusin-β reduces the level of the anti-inflammatory interleukin-1 receptor antagonist (IL-1Ra) [16]. Since IL-1Ra antagonizes the IL-1 receptor, its upregulation by salusin-α may represent a key element in modulating plaque-related inflammation [13]. In contrast, relatively few studies have examined the relationship between salusin-α and inflammatory processes in the vascular wall. Evidence suggests that salusin-α exerts anti-inflammatory effects at the site of atherosclerotic plaque formation. A key anti-atherogenic property of salusin-α is its ability to increase IL-1Ra expression, thereby suppressing the secretion of certain proinflammatory cytokines (IL-6, IL-8, and IL-18) [21]. Salusin-α also inhibits the synthesis of TNF-α, without affecting MCP-1 and VCAM-1 expression [15]. However, other studies have reported that salusin-α selectively reduces VCAM-1 expression, without significantly altering IL-6 and TNF-α expression [22]. Importantly, salusin-α has also been shown to alleviate oxidative stress by increasing the levels of antioxidant vitamins, particularly vitamin E and vitamin C, which further supports its protective role within the atherosclerotic environment [23].

These findings collectively support the notion that salusin-β acts as a potent proinflammatory mediator, while salusin-α exerts protective, anti-inflammatory effects within the atherosclerotic plaque microenvironment (Table 1).

## 4. The Role of Salusins in Foam Cell Formation

In vitro studies indicate that both types of salusins are involved in foam cell formation [24,26,28]. By activating the G-protein/c-Src/PKC/MAPK signaling pathway, salusins modulate the expression of acyl-CoA:cholesterol acyltransferase 1 (ACAT1) [3,24]. Salusin-β increases ACAT1 expression, thereby promoting the intracellular synthesis of cholesterol esters and accelerating foam cell formation [28,29]. Foam cell formation is critically dependent on scavenger receptors such as cluster of differentiation 36 (CD36), class A scavenger receptor (SR-A), and ATP-binding cassette transporter A1 (ABCA1). CD36 and SR-A primarily mediate the uptake of oxidized and acetylated low-density lipoprotein (LDL), whereas ABCA1 facilitates the efflux of excess cholesterol from foam cells in association with high-density lipoprotein (HDL) [31]. Studies in murine models have demonstrated that salusin-β increases the expression of CD36 and SR-A, but not that of ABCA1 [3]. Conversely, salusin-α inhibits ACAT1 activity and reduces CD36 expression [24,25,26,32]. However, other investigations in human cell cultures have not established any association between salusin-α and the expression of ABCA1 or SR-A [24,25].

Collectively, these findings highlight the divergent roles of salusin isoforms in macrophage-derived foam cell formation, with salusin-β enhancing and salusin-α attenuating this pivotal step in atherogenesis.

## 5. Salusins and the Proliferation of Vascular Smooth Muscle Cells and Fibroblasts

Several studies indicate that both salusin isoforms modulate the proliferation of vascular smooth muscle cells (VSMCs) and fibroblasts. However, salusin-β appears to play a more prominent role in these processes [1]. Salusin-β increases intracellular ionized calcium levels, which is a critical element in the signaling pathways that regulate the proliferation of fibroblasts and VSMCs [24]. It also stimulates the production of intracellular cyclic adenosine 3′,5′-monophosphate (cAMP), activating the cAMP-PKA-EGFR-CREB/ERK pathway, promoting vascular cell proliferation [3]. In vitro studies have demonstrated that salusin-β activates early response genes such as c-myc and c-fos, and by upregulating the mRNA expression of collagen I, collagen III, and fibronectin, it exacerbates VSMC fibrosis [27]. Notably, within atherosclerotic lesions of the coronary arteries, salusin-β immunoreactivity is strongly expressed in VSMCs and fibroblasts, in contrast to the minimal expression of salusin-α. Additionally, salusin-β promotes vascular wall fibrosis by activating the TGF-β1–Smad signaling pathway [27].

The role of salusin-α in VSMC and fibroblast proliferation is less clear. Some authors, including Shichiri et al. [1], suggest that salusin-α may also stimulate the proliferation of these cells, albeit to a lesser extent than salusin-β. Conversely, studies by Gao et al. [33] have reported that salusin-α inhibits VSMC proliferation via the Akt/mTOR pathway.

Further investigation is required to clarify whether the effects of salusin-α on vascular remodeling are context-dependent, influenced by the local microenvironment, or if they are disease-specific factors.

## 6. Influence of Salusins on Lipid Infiltration in Atherosclerotic Plaque

The accumulation of deposits composed of macrophages, LDL, foam cells, and cholesterol aggregates leads to the formation of the earliest atherosclerotic lesions, known as fatty streaks. In a subsequent phase, the accumulation of fibrous connective tissue results in the development of a mature atherosclerotic plaque [34]. Increased expression of salusin-β has been observed in both fatty streaks and in the deeper layers of the plaque, whereas salusin-α is present only in trace amounts [24].

Taken together, these findings suggest that salusin-β promotes atherogenic lipid infiltration, while salusin-α may exert a protective effect by attenuating lipid accumulation and foam cell development.

## 7. Plasma Salusin Levels and the Risk of Atherosclerotic Lesions

**Figure 1:**
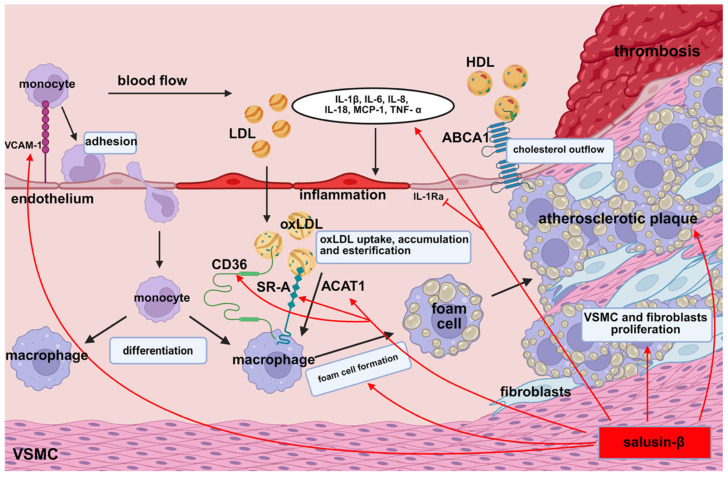
Mechanisms by which salusin-β promotes atherogenesis. ABCA1: ATP-binding cassette transporter A1, ACAT1: acyl-CoA/cholesterol acyltransferase 1, CD36: cluster of differentiation 36, HDL: high-density lipoprotein, IL-1β: interleukin-1β, IL-1Ra: interleukin-1 receptor antagonist, IL-6: interleukin-6, IL-8: interleukin-8, IL-18: interleukin-18, LDL: low-density lipoprotein, MCP-1: monocyte chemoattractant protein-1, oxLDL: oxidized low-density lipoprotein, SR-A: class A scavenger receptor, TNF-α: tumor necrosis factor α, VCAM-1: vascular cell adhesion molecule-1, VSMCs: vascular smooth muscle cells.

**Figure 2:**
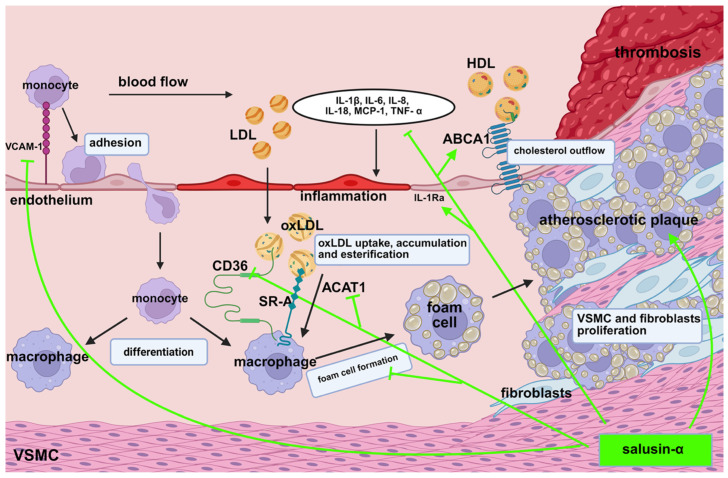
Mechanisms by which salusin-α protects against atherogenesis. ABCA1: ATP-binding cassette transporter A1, ACAT1: acyl-CoA/cholesterol acyltransferase 1, CD36: cluster of differentiation 36, HDL: high-density lipoprotein, IL-1β: interleukin-1β, IL-1Ra: interleukin-1 receptor antagonist, IL-6: interleukin-6, IL-8: interleukin-8, IL-18: interleukin-18, LDL: low-density lipoprotein, MCP-1: monocyte chemoattractant protein-1, oxLDL: oxidized low-density lipoprotein, SR-A: class A scavenger receptor, TNF-α: tumor necrosis factor α, VCAM-1: vascular cell adhesion molecule-1, VSMCs: vascular smooth muscle cells.

Recent clinical studies have demonstrated that elevated plasma levels of salusin-β or reduced levels of salusin-α are significantly correlated with the development of atherosclerotic lesions in blood vessels [27,35,36]. High plasma concentrations of salusin-β promote foam cell formation [24], elevate inflammatory factor levels, and accelerate plaque progression [15,18] (Figure 1). Lju J. et al. [37] suggest that reduced salusin-β expression may inhibit plaque development and could serve as a novel marker for cardiovascular disease progression. Conversely, increased expression of salusin-α appears to inhibit plaque development (Figure 2). These experimental findings are consistent with clinical observations indicating that patients with hypertension and coronary artery disease have significantly lower plasma levels of salusin-α compared to healthy individuals [35,38]. Furthermore, in patients with primary hypertension and carotid atherosclerotic changes, plasma levels of salusin-α are significantly lower than those observed in healthy populations [35,39]. These observations highlight salusin-α as a promising candidate biomarker for early detection and risk assessment in atherosclerotic cardiovascular disease.

Emerging evidence includes the so far unpublished study by Niepolski et al., which demonstrates a significant association between plasma salusin-α and various anthropometric measures [40]. The study includes six standard indicators: waist-to-hip ratio, waist-to-height ratio, body mass index, mid-upper arm muscle circumference (MUAMC), mid-upper arm circumference, and triceps skinfold thickness (TSF). The analysis revealed that higher plasma salusin-α concentrations positively correlated with muscle mass assessed by MUAMC and negatively correlated with subcutaneous fat measured by TSF.

## 8. Knowledge Gaps

One major unresolved issue is the precise mechanistic basis underlying the divergent biological effects of salusin-α and salusin-β in vascular inflammation and remodeling. Although both peptides originate from the same precursor, it remains unclear how their structural differences lead to opposing cellular and molecular actions. The existing literature presents conflicting data concerning the effects of salusin-α on VSMC proliferation, with some studies indicating a stimulatory effect [1] and others describing inhibitory actions mediated by specific intracellular signaling pathways [33]. Moreover, the influence of disease states and tissue-specific microenvironments on salusins activity is not yet fully understood, suggesting that their effects might vary significantly depending on specific physiological or pathological settings. Inconsistencies in reported plasma levels of salusin isoforms across patient populations and disease stages further complicate interpretation, potentially reflecting methodological variation or unrecognized regulatory mechanisms. Additionally, the relationship between salusins and established atherogenic processes, such as lipid metabolism and immune cell recruitment, has yet to be extensively investigated. Clarifying these mechanistic ambiguities and reconciling conflicting data are essential for substantiating the clinical validity of salusins as biomarkers or therapeutic targets in atherosclerosis. Future research should address these complexities to more accurately define the clinical significance and therapeutic potential of salusin peptides in cardiovascular disease.

## 9. Opportunities and Therapeutic Implications of Salusins

Emerging research highlights the promising therapeutic potential of targeting salusin peptides in the context of atherosclerotic cardiovascular disease. Salusin-α, recognized for its anti-inflammatory and anti-atherogenic effects, is considered a potential modulator of key vascular processes [3]. Preclinical studies suggest that enhancing salusin-α expression may help counteract crucial mechanisms in atherogenesis, such as endothelial dysfunction, inflammatory cytokine activity, lipid accumulation, foam cell formation, and the proliferation of vascular smooth muscle cells [30].

A study by Qian et al. demonstrated the efficacy of salusin-α gene transfer in a rabbit model of atherosclerosis. The intervention led to significant reductions in total cholesterol and LDL levels, and it effectively suppressed vascular intimal hyperplasia [26]. These findings indicate that increasing salusin-α levels may provide a dual benefit: modulating lipid metabolism and preventing pathological vascular remodeling. This positions salusin-α as a potential therapeutic agent with relevance throughout various stages of atherosclerotic disease.

In contrast, salusin-β exhibits potent proinflammatory, pro-atherogenic, and profibrotic properties, making it a compelling target for inhibition [41]. Experimental studies employing adenoviral vectors encoding salusin-β short hairpin RNA (shRNA) have shown that silencing salusin-β results in several favorable cardiovascular outcomes [42]. These include reduced arterial pressure, improved endothelium-dependent vasodilation, enhanced autonomic and cardiac function, and a measurable reduction in oxidative stress and systemic inflammation. The benefits appear to stem from the suppression of pathways involved in endothelial activation and plaque destabilization. These results support the therapeutic rationale for salusin-β antagonism as a novel anti-atherosclerotic strategy.

Taken together, the data point to a promising therapeutic paradigm based on modulating the salusins axis. Enhancing salusin-α signaling while suppressing salusin-β activity could provide synergistic benefits when integrated with current atherosclerosis treatment approaches.

In addition to their therapeutic viability, salusins are under investigation as potential biomarkers for disease diagnosis and prognosis. Circulating levels of both isoforms have been shown to correlate with the extent of vascular lesions, disease severity, and associated cardiovascular risk factors. Targeting salusin peptides using gene therapy, peptide analogs, or specific inhibitors may offer a new and effective strategy for treating and preventing atherosclerosis. However, the translation of these findings into clinical practice will require further validation through well-designed, large-scale studies. Similar to salusins, resistin is a well-known adipocytokine that participates in the regulation of metabolic and inflammatory processes and is implicated in the pathogenesis of cardiovascular disease [43]. Both types of peptides share functional roles in promoting vascular inflammation and endothelial dysfunction, suggesting their potential as important components in the future framework of cardiovascular therapeutics.

## 10. Summary

**Figure 3:**
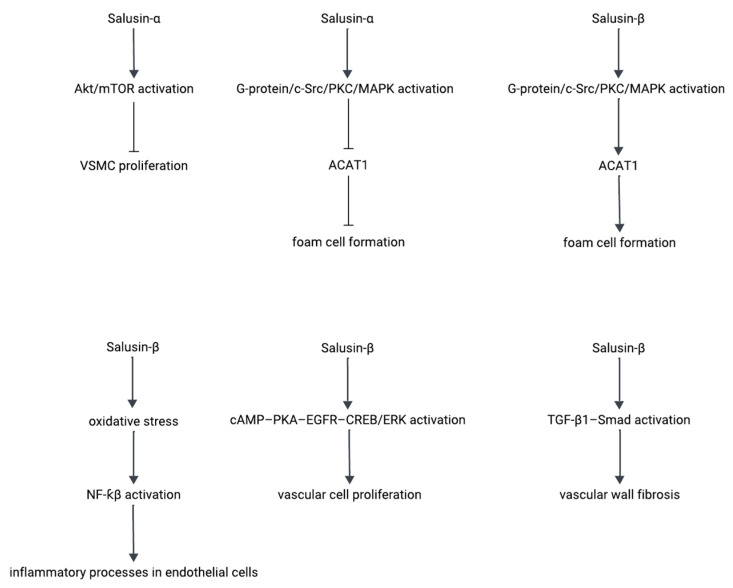
Signaling pathways mediating the pro-atherogenic and anti-atherogenic effects of salusin-α and salusin-β peptides. Arrows with a pointed head, which indicate activation or stimulation, and arrows with a flat end (without a head), which represent inhibition.

Salusin-α and salusin-β are two bioactive peptides generated from the same precursor but exhibiting contrasting effects on atherogenesis. Salusin-β promotes vascular inflammation by upregulating proinflammatory cytokines, enhancing foam cell formation, and accelerating the proliferation of vascular smooth muscle cells. In contrast, salusin-α has anti-inflammatory and anti-atherogenic effects by inhibiting key mediators of inflammation and foam cell development. Schematic diagrams of the signaling pathways involved in these processes are presented in Figure 3, illustrating their underlying molecular mechanisms.

This review details the biosynthesis and tissue expression of these peptides, their divergent impacts on endothelial dysfunction, foam cell formation, and vascular remodeling, and discusses the significance of plasma salusin levels as potential biomarkers for early detection of atherosclerotic cardiovascular disease. Further research is needed to confirm the diagnostic relevance of salusins and to better define their functional roles in the early development of atherosclerosis. Salusin appears to act as a novel factor linking metabolic disturbances, inflammatory responses, and the development of atherosclerosis.

## Figures and Tables

**Table 1:** Effects of salusin-α and salusin-β on atherosclerotic lesion development (↓—decrease, ↑—increase, →—no action/no data).

Action	Salusin-α	Citation	Material	Salusin-β	Citation	Material
Development of inflammatory processes	↓	[21]	HUVEC	↑	[16]	HUVEC
Activation of *NF-ƙB* pathway	→	[21]	HUVEC	↑	[15]	ApoE-/- mice
VCAM-1 expression	→/↓	[15,22]	ApoE-/- mice, HUVEC	↑	[16,18]	HUVEC
mRNA ICAM-1 expression	→	-	-	↑	[16,18]	HUVEC
IL-1β expression	→	-	-	↑	[3,18]	HUVEC
IL-6 expression	↓/→	[21,22]	HUVEC	↑	[16]	HUVEC
IL-8 expression	↓	[21]	HUVEC	↑	[16]	HUVEC
IL-18 expression	↓	[21]	HUVEC	↑	[16]	HUVEC
IL–1Ra expression	↑	[21]	HUVEC	↓	[16]	HUVEC
MCP-1 expression	→	[15]	ApoE-/- mice	↑	[3,15,18]	HUVEC; ApoE-/- mice
TNF-α expression	↓/→	[15,22]	ApoE-/- mice; HUVEC	↑	[19]	H9c2 or neonatal rat cardiomyocytes
Foam cel formation	↓	[24,25]	Human macrophages from monocytes	↑	[24,26,27]	Human macrophages from monocytes; atherosclerotic rabbit model; Human VSMCs
ACAT1 expression	↓	[24,25]	Human macrophages from monocytes	↑	[28,29]	Human VSMCs
CD36 expression	↓	[26,30]	Atherosclerotic rabbit model; ApoE-/- mice	↑	[30]	ApoE-/- mice
SR-A expression	→	[24,25]	Human macrophages from monocytes	↑/→	[3,24]	HUVEC; human macrophages from monocytes
ABCA1 expression	↑/→	[24,25,26]	Human macrophages from monocytes; Atherosclerotic rabbit model	→	[3,24]	HUVEC; human macrophages from monocytes
VSMC proliferation	↑/↓	[1]	Rat and human VSMCs	↑	[1]	Rat and human VSMCs
Fibroblast proliferation	↑/↓	[1]	Rat VSMCs	↑	[1]	Rat VSMCs
Presence in atherosclerotic plaque	↑	[24]	Human macrophages from monocytes	↑	[24]	Human macrophages from monocytes

Abbreviations: ABCA1, ATP-binding cassette transporter A1; ACAT1, acyl-CoA/cholesterol acyltransferase 1; ApoE-/- apolipoprotein E-deficient; CD36, class B scavenger receptor; HUVEC, human umbilical vein endothelial cell; ICAM-1, intercellular adhesion molecule-1; IL-1β, interleukin-1β; IL-1Ra, interleukin-1 receptor antagonist; IL-6, interleukin-6; IL-8, interleukin-8; IL-18, interleukin-18; MCP-1, monocyte chemoattractant protein-1; NF-ƙB, nuclear factor kappa-light-chain-enhancer of activated B cells (or simply nuclear factor kappa B); SR-A, class A scavenger receptor; TNF-α, tumor necrosis factor alpha; VCAM-1, vascular cell adhesion molecule-1; VSMCs, vascular smooth muscle cells.

## Data Availability

No new data were created or analyzed in this study. Data sharing is not applicable to this article.

## Abbreviations

The following abbreviations are used in this manuscript:

ABCA1	ATP-binding cassette transporter A1
ACAT1	Acyl-CoA, cholesterol acyltransferase 1
cAMP	3′,5′-Cyclic adenosine monophosphate
CD36	Cluster of differentiation 36
HDL	High-density lipoprotein
ICAM-1	High-density lipoprotein
IL-1β	Interleukin-1β
IL-1Ra	Interleukin-1 receptor antagonist
IL-6	Interleukin-6
IL-8	Interleukin-8
IL-18	Interleukin-18
LDL	Low-density lipoprotein
MCP-1	Monocyte chemoattractant protein-1
MUAMC	Mid-upper arm muscle circumference
SR-A	Class A scavenger receptor
TNF-α	Tumor necrosis factor α
TSF	Triceps skinfold thickness
VCAM-1	Vascular cell adhesion molecule-1
VSMCs	Vascular smooth muscle cells
